# Microwave-Assisted Synthesis of Carbon Nanospheres and Their Application as Plugging Agents for Oil-Based Drilling Fluids

**DOI:** 10.3390/molecules30030463

**Published:** 2025-01-21

**Authors:** Kaihe Lv, Haokun Shen, Yuan Geng, Mei-Chun Li, Hongyan Du, Xianbin Huang, Jinsheng Sun

**Affiliations:** 1 Department of Petroleum Engineering, China University of Petroleum (East China), Qingdao 266580, China; 2 Key Laboratory of Unconventional Oil & Gas, Development Ministry of Education, Qingdao 266580, China; 3 CNPC Engineering Technology R&D Company Ltd., Beijing 102206, China; 4 Co-Innovation Center of Efficient Processing and Utilization of Forest Resources, College of Materials Science and Engineering, Nanjing Forestry University, Nanjing 210037, China

**Keywords:** carbon nanospheres, plugging agent, oil-based drilling fluid, hydrothermal, microwave

## Abstract

Wellbore instability caused by the invasion of drilling fluids into formations remains a significant challenge in the application of oil-based drilling fluids (ODFs). In this study, carbon nanospheres (CNSs) were synthesized using glucose as the carbon source through a microwave-assisted method. The effects of the reaction temperature, carbon source concentration, and reaction time on the particle size of CNSs were systematically investigated. The results revealed that under optimal conditions, CNSs with an average particle size of 670 nm were successfully synthesized, exhibiting high sphericity and excellent dispersibility. CNSs demonstrated stable dispersion in mineral oil when lecithin was used as a dispersant. The plugging performance of CNSs in ODFs was evaluated through low-pressure filtration and high-temperature, high-pressure (HTHP) filtration tests. After aging at 180 °C for 16 h, the addition of 2% CNSs reduced the filtration volume from 10.6 mL to 2.5 mL on standard filter paper (average pore size: 3 μm) and from 8.5 mL to 1.6 mL on microporous membranes (average pore size: 0.5 μm). Additionally, the HTHP filtration volume decreased from 73 mL to 18 mL, and the permeability of the filter cake formed during HTHP filtration was reduced from 26.5 × 10^−3^ mD to 1.2 × 10^−3^ mD. Furthermore, CNSs improved the rheological properties and emulsion stability of ODFs. With excellent compatibility and applicability, CNSs offer a promising solution for enhancing the performance of oil-based drilling fluids.

## 1. Introduction

With the continuous rise in the global energy demand and the gradual depletion of shallow conventional oil and gas resources, the petroleum industry has increasingly shifted its focus toward the development of deep and ultra-deep oil and gas reservoirs [1]. Similarly, geothermal energy, as a promising alternative to fossil fuels, relies on drilling technologies analogous to those used for deep oil and gas wells [2]. However, the exploitation of these deep resources is often accompanied by high-temperature and high-pressure (HTHP) conditions, which impose stringent requirements on the performance of drilling fluids [3,4]. Under such extreme environments, the rheological properties, thermal stability, and plugging capabilities of drilling fluids play a decisive role in the success of drilling operations [5,6].

Under complex formation conditions, oil-based drilling fluids (ODFs) are preferred for high-temperature and high-pressure horizontal wells due to their excellent thermal stability, superior lubricating properties, and strong inhibition of shale hydration [7,8]. However, ODFs face persistent challenges in shale formations, particularly severe fluid loss issues. Shale formations are typically characterized by the presence of micro-fractures and nanoscale pores, which allow the filtrate from ODFs to infiltrate easily [9,10]. Such infiltration results in increased pore pressure, fracture connectivity, and propagation, leading to borehole collapse, severe fluid loss, and other downhole complications [11,12,13]. These issues significantly raise drilling costs and prolong operational timelines.

The selection and performance of plugging agents are critical for mitigating fluid loss in ODFs. Effective plugging agents form a dense plugging layer, preventing filtrate invasion, stabilizing borehole walls, and reducing fluid loss [14,15]. However, the application of conventional plugging agents under HTHP conditions faces considerable challenges. Current commercial plugging agents for ODFs primarily include silica-based materials and their derivatives [16], ultrafine calcium carbonate [17], resins [18,19], and asphalts [20]. Traditional ODF plugging agents often have large particle sizes, limiting their ability to penetrate the micro-fractures and nanopores of shale formations, thus reducing their plugging efficiency [21]. As filtrate invades these fractures, pore pressure rises, triggering fracture propagation and exacerbating borehole instability and fluid loss [22]. Asphalt-based materials are commonly used as plugging agents, but their application requires the softening point of the asphalt to match the formation temperature. In practice, determining the optimal softening point is challenging. Moreover, asphalt resins can negatively affect the rheological properties of ODFs, hindering the rate of penetration [23]. Similarly, high-molecular-weight polymeric nanoplugging agents, while offering some plugging effectiveness, often lack the thermal stability and high temperature resistance necessary for deep and ultra-deep well conditions [24,25,26,27].

Nanomaterials, due to their unique physicochemical properties, such as small size effect, high specific surface area, excellent thermal stability, and tunable surface chemistry, have demonstrated significant potential in improving the performance of ODFs. Studies have shown that nanomaterials can penetrate micro-fractures and nanopores, forming highly efficient plugging layers that prevent filtrate invasion and enhance borehole stability [28,29]. Furthermore, nanomaterials can improve the rheological and filtration properties of ODFs, making them suitable for complex operating conditions [30,31]. Carbon-based nanomaterials, such as multi-walled carbon nanotubes (MWCNTs) and graphene oxide, have been recognized as ideal additives for enhancing the performance of drilling fluids due to their exceptional thermal stability and high specific surface area [32,33,34]. Research indicates that these materials can effectively penetrate micro-fractures, forming plugging layers that improve both the plugging performance and thermal stability of ODFs. However, the widespread application of these materials is limited by their high cost and complex preparation processes. Additionally, systematic studies on the application of carbon-based nanomaterials in ODFs remain insufficient, particularly with respect to their plugging performance and underlying mechanisms under HTHP conditions. To address the limitations of traditional plugging agents in ODFs, this study explores the use of carbon nanospheres (CNSs) as a novel nanoplugging agent.

Microwave-assisted synthesis of CNSs has become a significant research focus in the field of carbon nanomaterials [35]. Compared to traditional heating methods, microwave heating offers higher energy conversion efficiency and more uniform heating, leading to notable advantages in improving synthesis efficiency, reducing reaction time, and enhancing product quality [36]. Microwave heating can rapidly and evenly heat the reaction system, typically completing the reaction within minutes, whereas traditional methods may require several hours. This significantly improves efficiency and reduces energy consumption. Additionally, the uniformity of microwave radiation ensures even temperature distribution, preventing temperature gradient issues common in conventional heating, which improves product consistency and stability. These features make microwave-assisted synthesis highly suitable for the large-scale industrial production of carbon nanomaterials [37]. Furthermore, microwave-assisted synthesis offers environmental benefits. Unlike traditional high-temperature sintering methods, it operates at lower temperatures and can use green solvents, reducing harmful emissions and environmental impact [38]. The method also allows for the precise control of the reaction time and conditions, minimizing byproduct formation and improving product purity and stability. The reduced byproducts and lower temperatures contribute to the environmental sustainability of the process [39,40]. The primary objective of this study is to systematically evaluate the plugging performance of microwave-assisted CNSs in ODFs, particularly under HTHP conditions.

In this study, glucose was used as a carbon source for synthesizing CNSs via microwave-assisted methods. Lecithin was subsequently employed to modify the surface of CNSs, enhancing their lipophilicity and compatibility with ODFs. The chemical structure, morphology, thermal properties, surface wettability, and dispersion stability of CNSs were characterized. A series of experiments were conducted to assess the plugging performance of the CNSs and their compatibility with ODFs. Additionally, the influence of CNSs on the rheological properties and emulsion stability of ODFs was investigated. The novelty of this study lies in the use of glucose as an eco-friendly and cost-effective carbon source for synthesizing CNSs, along with the microwave-assisted method, which significantly improves synthesis efficiency and reduces energy consumption compared to traditional methods. This work provides critical insights into the application of CNSs in ODFs, highlighting their potential to enhance plugging performance, rheological properties, and emulsion stability under challenging drilling conditions. However, challenges such as optimizing large-scale production and long-term stability under extreme downhole conditions remain to be addressed. This study offers valuable guidance for developing more efficient and environmentally sustainable drilling fluid technologies, with a particular focus on enhancing fluid loss control in complex drilling environments.

## 2. Results and Discussion

### 2.1. Optimization of Reaction Conditions of CNSs

Previous studies on the synthesis of CNSs using traditional hydrothermal methods have demonstrated that the reaction temperature, carbon source concentration, and reaction time significantly influence the morphology and yield of CNSs [41,42]. For the microwave-assisted hydrothermal synthesis of CNSs, these parameters were similarly investigated to evaluate their effects on the yield, particle size distribution, and morphology.

**Figure 1:**
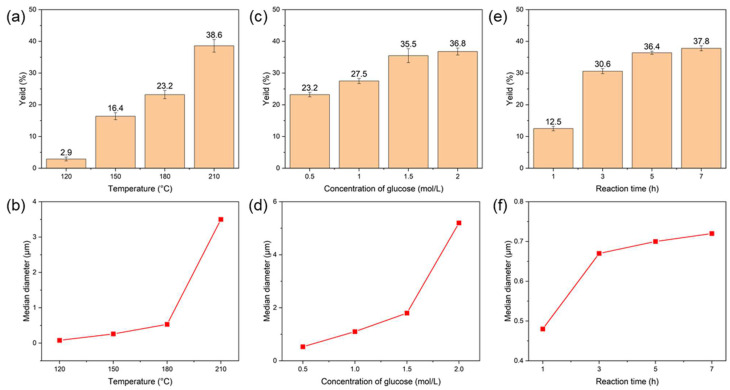
The effects of temperature (**a**,**b**), glucose concentration (**c**,**d**), and reaction time (**e**,**f**) on the yield and median particle size of CNSs, respectively.

**Figure 2:**
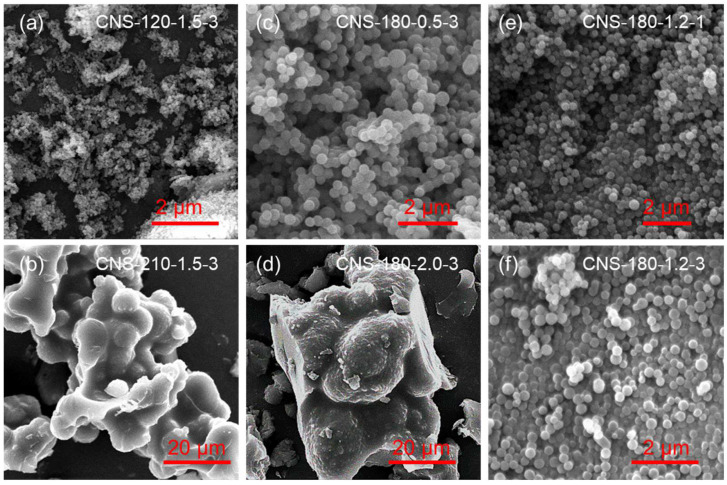
SEM images of CNSs synthesized under different conditions: (**a**) CNS-120-1.5-3, (**b**) CNS-210-1.5-3, (**c**) CNS-180-0.5-3, (**d**) CNS-180-2.0-3, (**e**) CNS-120-1.5-3, (**f**) CNS-180-1.2-3. These synthesized CNSs were labeled as CNS-*X*-*Y*-*Z*, where *X* indicates the synthesis temperature, *Y* represents the glucose concentration, and *Z* denotes the microwave reaction time.

The reaction temperature plays a critical role in the hydrothermal synthesis of CNSs, as it directly influences the dehydration and carbonization of glucose. As shown in Figure 1a,b, at a reaction temperature of 120 °C, the yield of CNSs was only 2.9%, with a median particle size of 0.08 μm. This temperature (120 °C) is generally too low to form carbon spheres in traditional hydrothermal methods. However, the rapid and uniform heating capability of the microwave-assisted hydrothermal method accelerated the dehydration and initial carbonization of the glucose, enabling CNS formation even at lower temperatures. The SEM image of CNS-120-1.5-3 (Figure 2a) shows small particle sizes with noticeable aggregation. When the temperature was increased to 180 °C, the yield significantly improved to 23.2%, and the median particle size increased to 0.53 μm. This indicates that higher reaction temperatures promote glucose dehydration and subsequent carbonization. Further increasing the temperature to 210 °C resulted in a yield of 38.6% and a sharp rise in the median particle size to 3.51 μm. The SEM images of CNS-210-1.5-3 (Figure 2b) showed substantial particle growth and agglomeration, suggesting that excessive temperatures accelerate the hydrothermal carbonization process, leading to larger particle sizes and reduced dispersibility. Therefore, 180 °C was determined to be the optimal reaction temperature, as it balanced high yield, good dispersibility, and uniform particle size.

The carbon source concentration determines whether polymer macromolecules in the reaction system can achieve supersaturation, thereby affecting the nucleation and growth rate of CNSs [43]. By fixing the reaction temperature at 180 °C and the reaction time at 3 h, glucose concentrations ranging from 0.5 mol/L to 2 mol/L were studied (Figure 1c,d). At a glucose concentration of 0.5 mol/L, the yield of CNSs was 23.2%, and the median particle size was 0.5 μm. The SEM image of CNS-180-0.5-3 revealed smooth particle surfaces, good dispersibility, and minimal aggregation (Figure 2c). This indicates that at low concentrations, polymer macromolecules in the solution are insufficient to reach supersaturation, leading to a limited number of nuclei and smaller, well-dispersed carbon spheres. Increasing the glucose concentration to 1.5 mol/L resulted in a higher yield (32.5%) and larger median particle size (1.2 μm), with the particles maintaining good dispersibility. This demonstrates that higher carbon source concentrations accelerate the supersaturation of polymer macromolecules, increasing the number of nuclei and the growth rate of carbon spheres. However, at 2 mol/L, the yield increased slightly to 36.8%, but the median particle size grew sharply to 5.2 μm, accompanied by significant aggregation (CNS-180-2.0-3) (Figure 2d). This can be attributed to excessive polymer macromolecules at high concentrations, which accelerate cross-linking between particles, leading to aggregation and non-uniform sizes [44]. Thus, 1.2 mol/L was chosen as the optimal glucose concentration, achieving a balance between a uniform particle size and high yield.

The reaction time also has a significant impact on the formation of CNSs, as it controls the extent of dehydration, polymerization, and carbonization reactions. By fixing a reaction temperature of 180 °C and a glucose concentration of 1.2 mol/L, the effect of the reaction time on CNS formation was evaluated (Figure 1e,f). The results showed that with longer reaction times, both the yield and particle size of CNSs gradually increased and eventually stabilized. After 1 h of reaction, the yield of CNSs reached 12.5%, and the median particle size was 0.48 μm, indicating partial dehydration and carbonization of glucose. Extending the reaction time to 3 h increased the yield to 30.6% and the median particle size to 0.67 μm. The SEM image confirmed that CNS-180-1.2-1 still maintained good dispersibility, with uniform particles and no noticeable aggregation (Figure 2e,f). When the reaction time was further extended to 7 h, both the yield and particle size growth plateaued, indicating that the reaction had nearly completed and that the growth rate of CNSs had significantly slowed. Considering reaction efficiency and energy costs, 3 h was selected as the optimal reaction time. Under this condition, the CNSs exhibited a high yield (30.6%), moderate particle size (0.67 μm), and excellent dispersibility.

In summary, the systematic optimization of the reaction temperature, carbon source concentration, and reaction time identified the optimal synthesis conditions as a reaction temperature of 180 °C, a glucose concentration of 1.2 mol/L, and a reaction time of 3 h. Under these conditions, the synthesized CNSs exhibited excellent dispersibility, uniform particle size (0.67 μm), and high yield (30.6%), making them suitable for further performance characterization and applications in ODFs.

### 2.2. Characterization of CNSs

**Figure 3:**
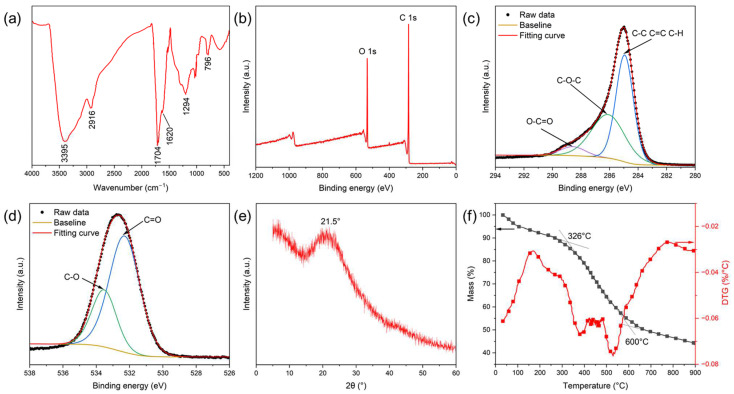
Characterization of CNSs: (**a**) FT-IR spectra; (**b**) XPS full spectra; (**c**) fine XPS spectra of C 1s; (**d**) fine XPS spectra of O 1s; (**e**) XRD pattern; (**f**) TG and DTG curves.

The FT-IR spectrum of the CNSs is shown in Figure 3a. The absorption peak at 3395 cm^−1^ corresponds to the stretching vibration of -OH groups, while the peak at 2916 cm^−1^ is attributed to the stretching vibration of -CH groups. The characteristic absorption peak at 796 cm^−1^ originates from the out-of-plane bending vibrations of aromatic and furan C-H groups. Additionally, the absorption peak at 1704 cm^−1^ corresponds to the stretching vibration of C=O groups, indicating the presence of carbonyl or carboxyl groups. The stretching vibration of C=C in the aromatic ring skeleton is observed at 1620 cm^−1^. The absorption peak at 1294 cm^−1^ corresponds to the stretching vibration of C-O-C groups. These absorption peaks are consistent with reports in the literature, indicating the presence of various active functional groups on the CNS surface [45]. This validates the successful synthesis of the CNSs. The chemical composition of the CNSs was further investigated using XPS. As shown in Figure 3b, the CNSs primarily contained carbon (C) and oxygen (O) elements, as evidenced by prominent C 1s and O 1s signals. The fine spectrum of the C 1s peak (Figure 3c) reveals an absorption peak at 288.6 eV, corresponding to carboxyl, ester, or lactone (-COOR); a peak at 286.2 eV, attributed to ether (-C-OR); and a peak at 284.9 eV, representing carbon-containing functional groups such as C=C/C-H/C-C. Similarly, the fine spectrum of the O 1s peak (Figure 3d) shows an absorption peak at 532.3 eV, corresponding to O=C groups, and another peak at 533.5 eV, representing C-O groups. The XRD pattern of the CNSs (Figure 3e) exhibits a broad peak at 21.5°, corresponding to an interlayer spacing (d_002_) of 0.411 nm, characteristic of the (002) plane of graphite structures. This broad peak indicates the amorphous carbon characteristics of the CNSs.

Figure 3f shows the results of the thermogravimetric analysis (TGA) and thermogravimetric derivative curves (DTGs) used to evaluate the thermal stability of the CNSs under a nitrogen atmosphere. The weight loss of the CNSs from 25 °C to 900 °C can be divided into four stages. Initially, the mass remained stable until a significant decrease began at approximately 200 °C, indicating thermal decomposition. The first major weight loss, occurring between 25 °C and 150 °C, was primarily due to the evaporation of adsorbed water on the surface. A stable plateau was observed between 150 °C and 326 °C, indicating good thermal stability. The significant weight loss between 326 °C and 600 °C corresponds to the thermal degradation of oxygen-containing functional groups (e.g., carboxyl, hydroxyl, and ether groups), with the maximum rate of mass loss observed at 526 °C. The DTG curve shows the highest mass loss rates at 326 °C and 600 °C, corresponding to different pyrolysis processes. Above 600 °C, the remaining carbon underwent continuous degradation until fully consumed. At 900 °C, the residual mass of the CNSs was 44.2%, demonstrating that their main carbon structure possessed high thermal stability.

### 2.3. Dispersion Stability of CNSs

**Figure 4:**
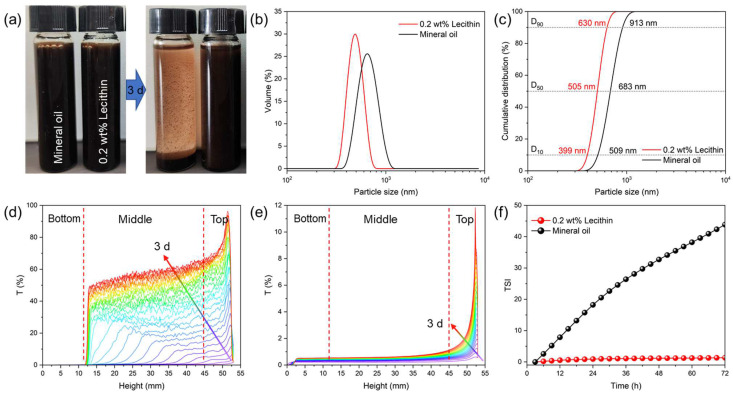
(**a**) Images of CNSs in mineral oil and 0.2 wt% lecithin before and after standing for 3 days. (**b**) The particle size differential distribution curve and (**c**) the cumulative distribution curve of CNSs in the mineral oil and 0.2 wt% lecithin solution. (**d**) Turbiscan LAB stability test for CNSs in mineral oil and (**e**) in 0.2 wt% lecithin solution. A line with arrows from violet to red represents a change from 0 to 3 days. (**f**) TSI value as a function over time.

Dispersion stability is a critical performance indicator for drilling fluid additives, as it directly affects their applicability in complex downhole environments. As shown in Figure 4a, when the CNSs were dispersed in mineral oil and left undisturbed for 3 days, the particles nearly completely settled at the bottom of the sample container, with only a small fraction remaining suspended in the solution. This result demonstrates poor dispersion stability in mineral oil, likely due to the incompatibility between the hydrophilic functional groups on the CNS surface and the low polarity of the mineral oil, which promotes particle agglomeration and sedimentation [46]. To improve the dispersion performance of the CNSs, 0.2 wt% lecithin was added to the mineral oil as a dispersant. Lecithin, a commonly used dispersant in ODFs, improves the compatibility of CNSs in oil-based systems without requiring additional processing. Its excellent interfacial activity enhances particle dispersion by modifying the wettability of the CNS surface. After adding 0.2% lecithin, the stability of the CNS dispersion in the mineral oil over 3 days was significantly improved.

From the particle size distribution curve (Figure 4c), it can be observed that the particle size of the CNSs in the lecithin solution ranged from 300 nm to 800 nm, with a more uniform and monodisperse distribution. The D_10_, D_50_, and D_90_ values were 399 nm, 505 nm, and 630 nm, respectively. In contrast, in the mineral oil without lecithin, the particle size of the CNSs increased significantly and exhibited a broader distribution. This indicates that lecithin effectively prevents particle agglomeration, thereby improving dispersion stability. To evaluate the stability of the CNSs in different solutions, multi-light-scattering technology was employed. Along the axis of the sample tube, the solution can be divided into bottom, middle, and top regions. As shown in Figure 4d, over 3 days, the intensity of the transmittance (T%) curve at the top of the sample gradually increased, with the high signal intensity extending toward the bottom of the sample container, reaching 12 mm after 3 days. By the end of the test, the transmittance at the top of the sample approached 100%, indicating significant sedimentation of the CNSs in the mineral oil. In contrast, in the 2% lecithin solution, the transmittance changes in the CNS suspension were significantly reduced (Figure 4e). Only the top 5 mm region showed a slight increase in transmittance, with a maximum value of about 12%, which is negligible compared to the CNSs in the mineral oil. This demonstrates that lecithin significantly suppresses CNS sedimentation in mineral oil, effectively improving its dispersion stability. The TSI is a simple and convenient parameter that reflects the magnitude of particle concentration changes throughout the test. A larger TSI value indicates a less stable system. As shown in Figure 4f, the TSI value of the CNSs in the mineral oil without lecithin gradually increased over time, indicating worsening sedimentation and decreasing dispersion stability. In contrast, with the addition of lecithin, the TSI value remained stable throughout the test, further confirming the significant improvement in CNS dispersion performance due to lecithin.

**Figure 5:**
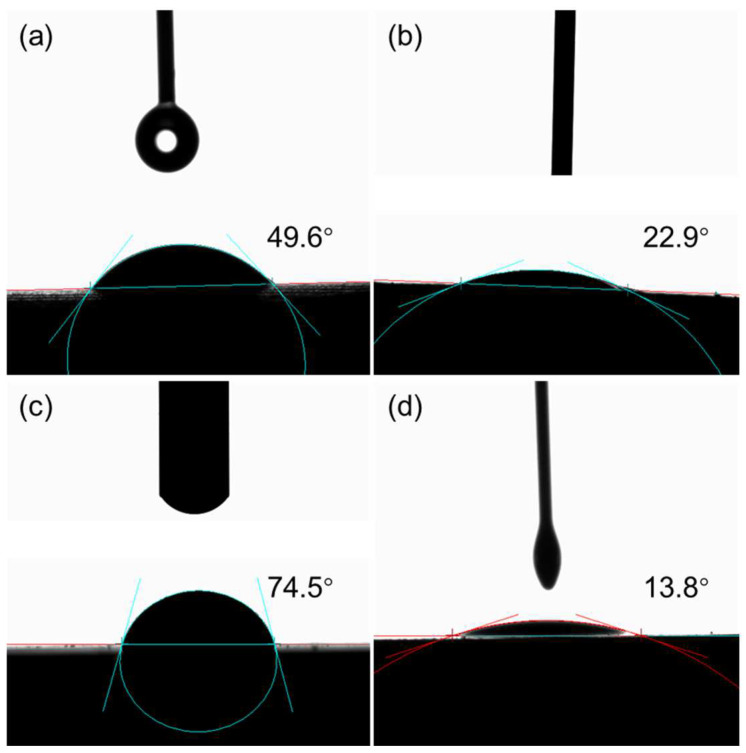
The contact angles of water and mineral oil on CNSs that separated from mineral oil (**a**,**b**) and the lecithin solution (**c**,**d**). During the testing process, the volume of the liquid was fixed at 3 μL to avoid the influence of the liquid volume on the droplet morphology.

It is well known that the dispersion stability of particles in a solvent is closely related to their surface wettability. To analyze the surface wettability of the CNSs, contact angle measurements were performed (Figure 5). The results indicate that the untreated CNS surface exhibited contact angles of 49.6° for water and 22.9° for oil, indicating moderate hydrophilicity and oleophilicity. However, after lecithin treatment, the contact angle for water significantly increased to 74.5°, while the contact angle for oil decreased to 13.8°. These changes suggest that the adsorption of lecithin molecules on the CNS surface made it more hydrophobic and more oleophilic. This wettability alteration enhances the compatibility of CNSs with mineral oil, thereby improving their dispersion performance.

### 2.4. Drilling Fluid Performance

The plugging performance of ODF is critical for maintaining wellbore stability and preventing fluid invasion into formations. Additionally, the rheological properties of ODF directly influence its ability to carry cuttings and the efficiency of borehole cleaning. In this study, the plugging performance of CNSs in ODF, as well as their effects on the rheological properties and emulsion stability, were systematically analyzed.

**Figure 6:**
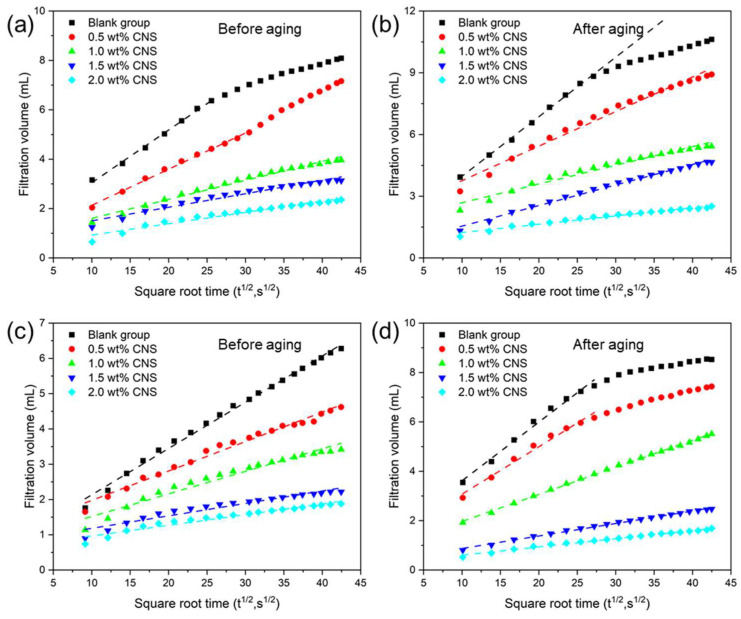
The filtration volume of ODF containing different concentrations of CNSs before and after aging in API standard filter paper (**a**,**b**) and PTFE microporous membranes (**c**,**d**) as a function of the square root of time.

According to API standards, the plugging performance of CNSs in ODF was evaluated. Figure 6a,b show the filtration behavior of ODF containing CNSs, both before and after aging, using standard filter paper with an average pore size of 3 μm. The filtration volume of the drilling fluid was proportional to the square root of time, consistent with our previous research [23]. As the CNS concentration increased from 0% to 2.0%, the filtration volume of the drilling fluid over 30 min decreased significantly. Before aging, the filtration volume decreased from 8.1 mL to 2.3 mL, while after aging, it decreased from 10.6 mL to 2.5 mL. This indicates that the CNSs effectively reduced the filtration volume and enhanced the plugging performance of the ODF. Similarly, the filtration behavior of the ODF was analyzed using a microporous filter medium with a pore size of 0.5 μm (Figure 6c,d). Due to the smaller pore size, the filtration volume further decreased. Before aging, the filtration volume dropped from 6.3 mL to 1.8 mL, and after aging, it decreased from 8.5 mL to 1.6 mL. These results demonstrate that the CNSs exhibited more pronounced plugging effects in smaller-pore-size media. Notably, for the ODF without CNSs, the slope of the filtration volume versus the square root of time gradually decreased. According to Darcy’s law, this slope is related to the permeability of the filter medium. A decreasing slope indicates that the pores in the filter medium are gradually being sealed, thereby reducing permeability. For the ODF with CNSs, the filtration volume increased linearly with the square root of time. This behavior can be attributed to the nanoscale CNS particles, which quickly penetrated and filled the nanopores of the filter cake under pressure, reducing the permeability early in the filtration process and significantly decreasing the filtration volume.

**Figure 7:**
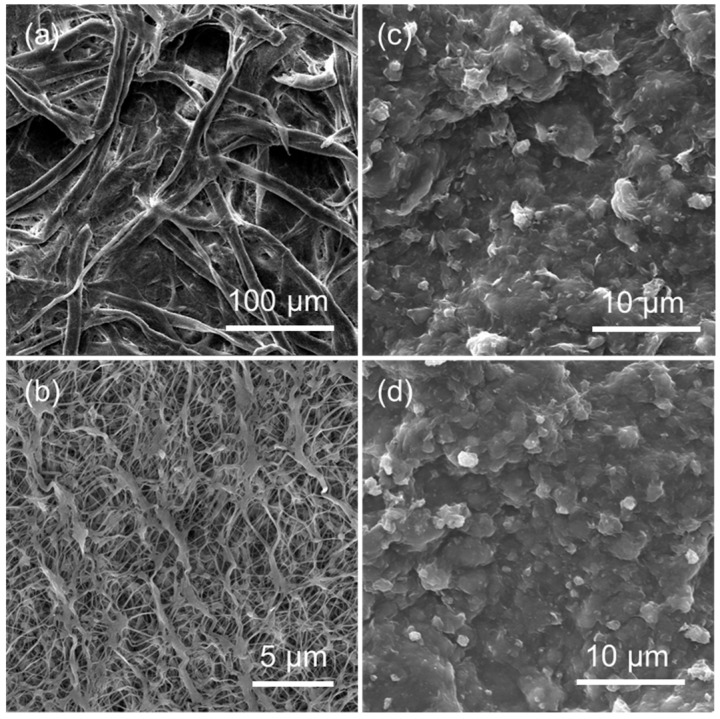
SEM images of API standard filter paper (**a**) before and (**b**) after filtration, and PTFE microporous membranes (**c**) before and (**d**) after filtration.

Microscopic morphology observations (Figure 7a,b) revealed that the filter media with a pore size of 2 μm had relatively large pores, with some reaching up to 70 μm in diameter, while the pore size of the 0.5 μm media was noticeably smaller. After the filtration experiment, clay particles and CNSs in the drilling fluid stacked to form a dense filter cake (Figure 7c,d), with no visible pores. This dense structure effectively prevented the infiltration of drilling fluid, ensuring wellbore stability.

**Figure 8:**
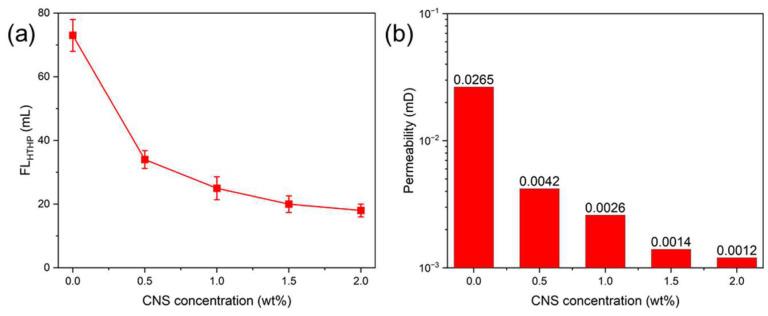
The HTHP filtration volume (**a**) and the permeability of filter cakes (**b**) of ODFs with different concentrations of CNSs.

Considering that ODFs are typically used in HTHP environments, the plugging performance of the CNSs was also evaluated under HTHP conditions. Compared to the filtration volume at 0.7 MPa, the filtration volume increased significantly under HTHP conditions. As the CNS concentration increased from 0% to 1.5%, the HTHP filtration volume decreased from 73 mL to 20 mL, indicating that the CNSs maintained excellent plugging performance under HTHP conditions (Figure 8a). Further increasing the CNS concentration to 2.0% resulted in an additional reduction in the HTHP filtration volume, though the effect was less pronounced. Additionally, analysis of the permeability of filter cakes formed during the HTHP filtration test showed that the CNSs significantly reduced the filter cake permeability. When the CNS concentration increased from 0% to 2.0%, the permeability of the filter cake decreased from 26.5 × 10^−3^ mD to 1.2 × 10^−3^ mD (Figure 8b). This indicates that the CNSs effectively filled most of the pores in filter cake, preventing the invasion of drilling fluid into the formation.

**Figure 9:**
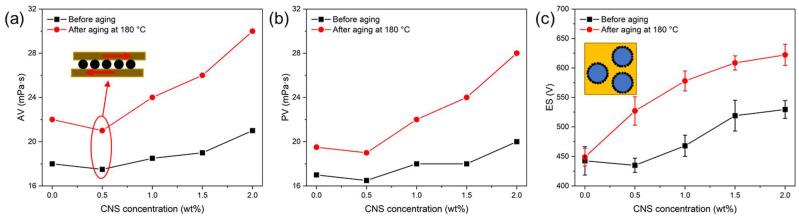
The influence of CNSs on the performance of ODFs before and after aging: (**a**) AV, (**b**) PV, and (**c**) ES.

Rheological performance is a critical parameter of drilling fluid, as it directly affects the suspension of cuttings and the efficiency of borehole cleaning. As shown in Figure 9a,b, the AV and PV of the drilling fluid increased with the rising CNS concentration. This can be attributed to the increased solid-phase content in the dispersion, which enhanced flow resistance due to solid–solid, solid–liquid, and liquid–liquid interactions. The increase in viscosity improved the ability to carry cuttings. Interestingly, when 0.5% CNS was added, both the AV and PV slightly decreased. This behavior may be attributed to the “ball-bearing effect” of CNSs, where CNSs reduce interparticle friction by converting sliding friction to rolling friction, thereby lowering flow resistance [47,48,49]. This phenomenon provides valuable insights for designing solid lubricants in ODFs.

The ES value is another key parameter of ODFs. As shown in Figure 9c, adding 2% CNS increased the ES value of the ODFs from 442.5 V to 529.5 V before aging and further increased it to 622.2 V after aging. This indicates that the CNSs significantly enhanced the emulsion stability of the ODFs. The improved stability is primarily attributed to the amphiphilic wettability of CNSs (Figure 5), which allows the CNS particles to adsorb at the oil–water interface, forming a stable particle film that prevents droplet coalescence. Thus, CNSs act as a solid emulsifier, demonstrating excellent compatibility with ODFs.

In summary, CNSs significantly enhance the plugging performance, rheological properties, and emulsion stability of ODFs. In terms of the plugging performance, CNSs effectively reduce the filtration volume by filling filter cake pores and lowering permeability, maintaining excellent performance even under HTHP conditions. Specifically, the addition of 2% CNS reduced the filtration volume from 10.6 mL to 2.5 mL on standard filter paper and from 8.5 mL to 1.6 mL on microporous membranes, which is a notable improvement compared to typical ODF formulations reported in the literature. In terms of rheological properties, CNSs increase the viscosity by 30%, reducing interparticle friction through the ball-bearing effect, which is in line with the improvements observed in similar studies using other nanomaterials. In terms of emulsion stability, the amphiphilic surface of the CNSs formed a stable structure at the oil–water interface, greatly enhancing emulsion stability. Under standard conditions, the addition of 1% CNS improved the emulsion stability by 25% compared to conventional ODF. These results highlight the potential of CNSs as a multifunctional additive in ODFs, demonstrating significant improvements over conventional additives and confirming their practical applicability in drilling fluid formulations.

## 3. Materials and Methods

### 3.1. Materials

Anhydrous glucose, calcium chloride (CaCl_2_), and aluminum chloride hexahydrate (AlCl_3_·6H_2_O) were procured from Shanghai Aladdin Biochemical Technology Co., Ltd. (Shanghai, China). Deionized water was prepared in the laboratory. The primary emulsifier, secondary emulsifier, organic clay, filtrate reducer, lecithin, lime, and mineral oil were supplied by CNPC Engineering Technology R&D Company Ltd. (Dubai, United Arab Emirates). The main components of the main emulsifier and sub emulsifier used were fatty acid derivatives, the organic clay was a quaternary ammonium cationic surfactant-modified montmorillonite, and the filtrate reducer was oxidized asphalt. Phospholipids were re used as solid dispersants. The kinematic viscosity and density of the mineral oil were 3 mPa·s and 0.81 g/cm^−3^, respectively, at 20 °C and 0.1 MPa.

### 3.2. Preparation of Carbon Nanospheres

**Figure 10:**
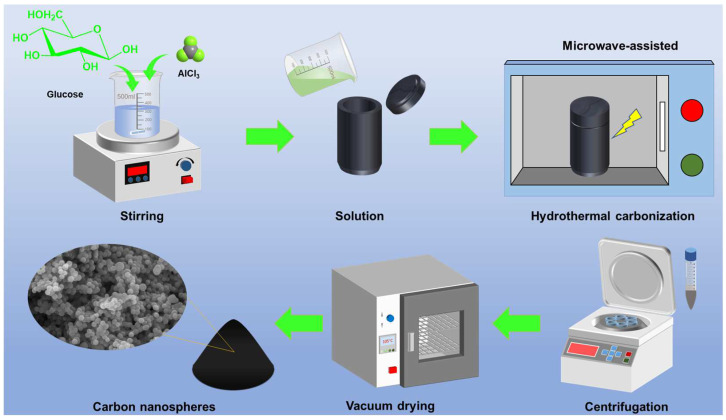
Schematic diagram of the preparation process of carbon nanospheres.

In a typical synthesis process, 18.02 g of glucose and 4.79 g of AlCl_3_ were dissolved in 100 mL of deionized water. A 50 mL portion of the solution was transferred into a 100 mL microwave tube and sealed. The solution was subjected to microwave-assisted hydrothermal carbonization at 180 °C for 3 h, with a maximum power of 800 W, using a microwave hydrothermal synthesis system (Nanocube XH-800SP, Beijing Xianghu Technology Development Co., Ltd., Beijing, China). After the reaction, the resulting dispersion was centrifuged, washed with deionized water and ethanol, and dried in a vacuum oven at 105 °C for 24 h to obtain the CNSs. The schematic diagram of the preparation process of the CNSs is shown in Figure 10. These synthesized CNSs were labeled as CNS-*X*-*Y*-*Z*, where *X* indicates the synthesis temperature (°C), *Y* represents the glucose concentration (mol/L), and *Z* denotes the microwave reaction time (h).

The yield of CNSs was calculated using Formula (1):

(1) $$\mathrm{Yield}=\frac{\mathrm{Actual}\;\mathrm{yield}}{\mathrm{Theoretical}\;\mathrm{yield}}\times 100\%$$

### 3.3. Preparation of ODFs

The ODFs were prepared following the formula and procedures outlined in Table 1.

### 3.4. Characterization Methods

The Fourier-transform infrared (FT-IR) spectrum of the CNSs was obtained using an FT-IR spectrometer (IRTracer-100, Shimadzu Corporation, Kyoto, Japan). The FT-IR spectra were recorded in the range 4000–400 cm^−1^ with a resolution of 4 cm^−1^.

Thermogravimetric analysis (TGA) was performed using a thermal analyzer to evaluate the thermal stability of the CNSs in the temperature range of 25–900 °C at a heating rate of 10 °C/min under a nitrogen atmosphere.

The X-ray diffraction (XRD) pattern of the CNSs was recorded using a Panalytical PRO PW3040/60 diffractometer (Almelo, The Netherlands) over 5° < 2θ < 60° range to characterize the crystalline structure of the CNSs. The interlayer spacing was calculated using Bragg’s equation.

The elemental composition of the CNSs was analyzed using an X-ray photoelectron spectroscopy (XPS) analyzer (KRATOS Axis Ultra, Kratos Analytical, Manchester, UK) equipped with an Al target (15 kV, 10 mA).

The particle size distribution of the CNSs was measured at 25 °C using a Zetasizer Nano Z (Malvern Instruments Co., Ltd., Malvern, UK) with a 90° detection angle.

Air–liquid contact angles were measured by an OCA-25 optical contact angle measuring instrument (DataPhysics Instruments Co., Ltd., Filderstadt, Germany). Dried CNS samples were pressed into thin sheet, and a water or oil droplet (3 μL) was placed on the surface. The droplet shape was immediately captured using an optical contact angle measurement instrument, and the contact angle was determined to evaluate the surface wettability of the CNSs.

### 3.5. Dispersion Stability Test

For dispersion stability experiments, 1 g of CNSs was dispersed in 100 mL of a mixture of mineral oil and lecithin (200:3, m/m). The mixture was subjected to high-shear mixing at 10,000 rpm for 20 min, followed by ultrasonic dispersion for 10 min. The suspension was allowed to stand for 72 h. The static stability of the suspension was quantitatively measured using a Turbiscan LAB instrument. Similarly, a blank control group was prepared by dispersing 1 g of CNSs in 100 mL of mineral oil without lecithin.

The Turbiscan LAB measures backscattered light as a function of distance along the sample tube and time through synchronous optical sensors. The embedded technology is based on multi-light scattering principles. Each phenomenon can be detected and quantified using backscattering (BS) and transmission (T) signals measured by the TURBISCAN technology, as both signals depend on the particle concentration and particle size.

To conduct an objective comparison of stability, the overall instability of the system must be considered. This means that the magnitude of instability throughout the entire sample must be quantitatively compared. Thus, the TURBISCAN Stability Index (TSI) is introduced; it is a robust, objective, and global parameter that can be determined with a single click [50]. This parameter accounts for the total instability and reflects the overall stability of a given sample. The TSI corresponds to the cumulative sum of all backscattering or transmission changes across the entire sample caused by instability. The smaller the value, the more stable the sample. The TSI was calculated using Formula (2):

(2) $$\mathrm{TSI}=\frac{1}{H}\sum_{ti=1}^{t\max}\sum_{zi=z\min}^{z\max}\left|BST(ti,tz)-BST(ti-1,tz)\right|$$

### 3.6. Oil-Based Drilling Fluid Performance Testing

Low-pressure filtration test: The plugging performance of ODF was evaluated using filtration experiments according to the American Petroleum Institute drilling fluid laboratory test standard [51]. API standard filter paper (average pore size: 3 μm) and polytetrafluoroethylene (PTFE) microporous membranes (average pore size: 0.45 μm) were employed as filtration media to simulate the micropores and nanopores typically present in formations. A 250 mL drilling fluid sample was subjected to filtration under a pressure of 0.75 MPa for 30 min, and the filtrate volume was recorded. To assess the effects of temperature, the ODF was aged at 150 °C for 16 h in a hot-rolling furnace (BGRL-5; Qingdao Tongchun, Qingdao, China), after which the filtration test was performed.

HTHP filtration: The filtration performance of the drilling fluid under HTHP conditions was evaluated by measuring the filtration volume over 30 min at 150 °C and 3.50 MPa. After the initial filtration test, the filter cake formed on the filtration medium was retained, and the drilling fluid was replaced with mineral oil. A second filtration experiment was conducted under the same conditions, and the final thickness of the filter cake was measured. Using Darcy’s law, the permeability of the filter cake was calculated from the recorded data [52].

Rheological properties: The rheological properties of the ODF were tested before and after aging using a rotational viscometer (ZNN-D6; Qingdao Haitongda Co., Ltd., Qingdao, China) at 65 °C. The apparent viscosity (AV), plastic viscosity (PV), and yield point (YP) were calculated using Equations (3) and (4):

(3) $$\mathrm{AV}=0.5\times \theta_{600}\;(\mathrm{mPa}\cdot s)$$

(4) $$\mathrm{PV}=\theta_{600}-\theta_{300}\;(\mathrm{mPa}\cdot s)$$

where *θ*_600_ and *θ*_300_ represent viscometer readings at 600 and 300 rpm, respectively.

Emulsion stability: The emulsion stability (ES) of the ODF was evaluated using an electric stability tester (DWY-2, Qingdao Tongchun Petroleum Instruments Co., Ltd., Qingdao, China) at 25 °C. During the test, the electrodes were immersed in the sample, and the voltage was gradually increased until emulsion breakdown occurred. The breakdown voltage was recorded to assess the emulsion’s stability.

## 4. Conclusions

In this study, CNSs were synthesized using glucose as a carbon source and AlCl_3_ as a catalyst under microwave irradiation. The optimal synthesis conditions (800 W microwave power, 180 °C reaction temperature, 1.2 mol/L glucose concentration, and 3 h reaction time) resulted in CNSs with excellent sphericity and dispersibility. When incorporated into ODFs, the CNSs significantly reduced the filtration volume and rate, particularly under HTHP conditions, and formed denser filter cakes, effectively reducing fluid loss. At low concentrations, the CNSs reduced the flow resistance, while at higher concentrations, they enhanced the viscosity, improving the ability to carry cuttings. Moreover, the CNS particles improved the emulsion stability of ODFs by adsorbing at the oil–water interface and forming stable particle films. CNSs demonstrated excellent compatibility with ODFs, making them a promising candidate for enhancing the performance of high-performance ODFs. However, challenges remain in optimizing the synthesis process for large-scale production and ensuring the long-term stability of CNSs under extreme downhole conditions. Future work should focus on overcoming these challenges to fully realize the potential of CNSs in industrial applications.

## Figures and Tables

**Table 1:** Preparation process and formula of the ODFs.

Addition Order	Additives	Dosage	Stirring Speed (rpm)	Stirring Time (min)
1	Mineral oil	255 mL	/	/
2	Primary emulsifier	10.0 g	10,000	10
3	Secondary emulsifier	8.0 g	10,000	10
4	Lecithin	0.6 g	10,000	10
5	20 wt% CaCl_2_ solution	45 mL	10,000	20
6	CaO	9.0 g	10,000	20
7	Organic clay	6.0 g	10,000	20

## Data Availability

The original contributions presented in this study are included in the article. Further inquiries can be directed to the corresponding author.
